# High-level expression of protein tyrosine phosphatase non-receptor 12 is a strong and independent predictor of poor prognosis in prostate cancer

**DOI:** 10.1186/s12885-019-6182-3

**Published:** 2019-10-12

**Authors:** Sören A. Weidemann, Charlotte Sauer, Andreas M. Luebke, Christina Möller-Koop, Stefan Steurer, Claudia Hube-Magg, Franziska Büscheck, Doris Höflmayer, Maria Christina Tsourlakis, Till S. Clauditz, Ronald Simon, Guido Sauter, Cosima Göbel, Patrick Lebok, David Dum, Christoph Fraune, Simon Kind, Sarah Minner, Jakob Izbicki, Thorsten Schlomm, Hartwig Huland, Hans Heinzer, Eike Burandt, Alexander Haese, Markus Graefen, Asmus Heumann

**Affiliations:** 1 Institute of Pathology, University Medical Center Hamburg-Eppendorf, Martinistrasse 52, 20246 Hamburg, Germany; 2 General, Visceral and Thoracic Surgery Department and Clinic, University Medical Center Hamburg-Eppendorf, Martinistrasse 52, 20246 Hamburg, Germany; 3 Department of Urology, Charité - Universitätsmedizin Berlin, Charitéplatz 1, 10117 Berlin, Germany; 4 Martini-Clinic, Prostate Cancer Center, University Medical Center Hamburg, Eppendorf, Germany

**Keywords:** PTPN12, Prostate cancer, Prognosis, Immunohistochemistry

## Abstract

**Background:**

Protein tyrosine phosphatase non-receptor 12 (PTPN12) is ubiquitously tyrosine phosphatase with tumor suppressive properties.

**Methods:**

PTPN12 expression was analyzed by immunohistochemistry on a tissue microarray with 13,660 clinical prostate cancer specimens.

**Results:**

PTPN12 staining was typically absent or weak in normal prostatic epithelium but seen in the majority of cancers, where staining was considered weak in 26.5%, moderate in 39.9%, and strong in 4.7%. High PTPN12 staining was associated with high pT category, high classical and quantitative Gleason grade, lymph node metastasis, positive surgical margin, high Ki67 labeling index and early prostate specific antigen recurrence (*p* < 0.0001 each). PTPN12 staining was seen in 86.4% of TMPRSS2:ERG fusion positive but in only 58.4% of ERG negative cancers. Subset analyses discovered that all associations with unfavorable phenotype and prognosis were markedly stronger in ERG positive than in ERG negative cancers but still retained in the latter group. Multivariate analyses revealed an independent prognostic impact of high PTPN12 expression in all cancers and in the ERG negative subgroup and to a lesser extent also in ERG positive cancers. Comparison with 12 previously analyzed chromosomal deletions revealed that high PTPN12 expression was significantly associated with 10 of 12 deletions in ERG negative and with 7 of 12 deletions in ERG positive cancers (*p* < 0.05 each) indicating that PTPN12 overexpression parallels increased genomic instability in prostate cancer.

**Conclusions:**

These data identify PTPN12 as an independent prognostic marker in prostate cancer. PTPN12 analysis, either alone or in combination with other biomarkers might be of clinical utility in assessing prostate cancer aggressiveness.

## Background

With more than 1.3 million estimated new cases worldwide in 2018, prostate cancer is the most common cancer in males in over one-half of the countries of the world [1]. The clinical course is highly variable. In elderly and symptom-free patients watchful waiting and active surveillance are alternatives to surgical therapy in localized disease [2]. The currently available criteria used for the distinction between high risk and low risk patients, such as Gleason grade, clinical stage and prostate specific antigen (PSA) level, are statistically powerful but not sufficient to enable optimal treatment decisions for every patient. To more reliably prevent unnecessary treatments better prognostic markers are needed.

Protein tyrosine phosphatase non-receptor 12 (PTPN12) is a member of the protein tyrosine phosphatases family, which is ubiquitously expressed [3, 4]. It dephosphorylates cellular tyrosine kinases, such as HER2 [5] and functions as a tumor suppressive key regulator of signaling pathways involved in cell-extracellular matrix crosstalk, cellular responses to mechanical stress and cell adhesion [6, 7]. The oncogene c-ABL is an important target of PTPN12 driven dephosphorylation resulting in its down regulation [8, 9]. A number of studies have reported that decreased expression of PTPN12 as determined by immunohistochemistry was found to be significantly associated with advanced tumor stage in hepatocellular [10, 11], renal cell [12], and urinary bladder [13] as well as in squamous cell carcinoma of the oral cavity, esophagus and nasopharynx [14–17]. High PTPN12 expression was described to be linked with favorable survival duration in non-small cell lung carcinoma patients [18] and with response to neoadjuvant chemotherapy in triple negative breast cancer [19].

Evidence suggests that PTPN12 expression might also be relevant for prostate cancer. Using PC-3 cell lines Sahu et al. showed a role of PTPN12 in regulating migration of prostate cells [20]. For this purpose, a preexisting prostate cancer tissue microarray (TMA) consisting of more than 13,000 prostate cancers with clinical follow-up information and attached molecular data was examined for PTPN12 expression levels.

## Methods

### Patients

The 13,660 patients had radical prostatectomy between 1992 and 2015 (Department of Urology and the Martini Clinic at the University Medical Center Hamburg-Eppendorf). Classical Gleason categories and “quantitative” Gleason grading was performed as described [21]. In brief, for quantitative Gleason grading the percentage of Gleason 4 patterns was recorded to categorize the Gleason grades in 12 groups. Follow-up was available for 12,208 patients with a median follow-up of 49 months (Table 1). PSA recurrence was defined as the time point when postoperative PSA level was ≥0.2 ng/ml. The TMA was produced with a single 0.6 mm core taken from a tumor containing tissue block for each patient [22]. The attached molecular database included data on Ki67 labeling index (Ki67LI) [23], HER2 immunostaining [24], ERG expression and ERG rearrangement analysis by fluorescence in situ hybridization (FISH) [25, 26], as well as deletion status of 5q21 (CHD1) [27], 6q15 (MAP3K7) [28], 10q23 (PTEN) [29], 3p13 (FOXP1) [30], 13q14 [31], 18q21 [32], 8p21 [33], 12p13 [34], 12q24 [35], 16q24 [36] and 17p13 [37]. Furthermore, data from deletions of 5q13 (5441 tumors, unpublished) were available.

**Table 1:** Pathological and clinical data of the arrayed prostate cancers

	No. of patients (%)	
	Study cohort on TMA^a^	Biochemical relapse among categories
Follow-up		
n	12,208	2759 (22.6%)
Mean / median (month)	59 / 49	–
Age (y)		
≤ 50	310	54 (17.4%)
51–59	3278	656 (20.0%)
60–69	7539	1693 (22.5%)
≥ 70	2251	501 (22.3%)
Pretreatment PSA (ng/ml)		
< 4	1659	242 (14.6%)
4–10	7942	1355 (17.1%)
10–20	2807	737 (26.3%)
> 20	940	397 (42.2%)
pT stage (AJCC 2002)		
pT2	8646	1095 (12.7%)
pT3a	2904	817 (28.1%)
pT3b	1765	796 (45.1%)
pT4	68	51 (75%)
Gleason grade		
≤ 3 + 3	2638	264 (10.0%)
3 + 4	7172	1436 (20.0%)
3 + 4 Tert.5	645	165 (25.6%)
4 + 3	1224	683 (55.8%)
4 + 3 Tert.5	987	487 (49.3%)
≥ 4 + 4	756	531 (70.2%)
pN stage		
pN0	7899	1821 (23.1%)
pN+	855	546 (63.9%)
Surgical margin		
Negative	10,768	1833 (17.0%)
Positive	2613	1059 (40.5%)

*Abbreviation: AJCC*, American Joint Committee on Cancer

^a^ Numbers do not always add up to 13,660 in the different categories because of cases with missing data

### Immunohistochemistry (IHC)

**Fig. 1:**
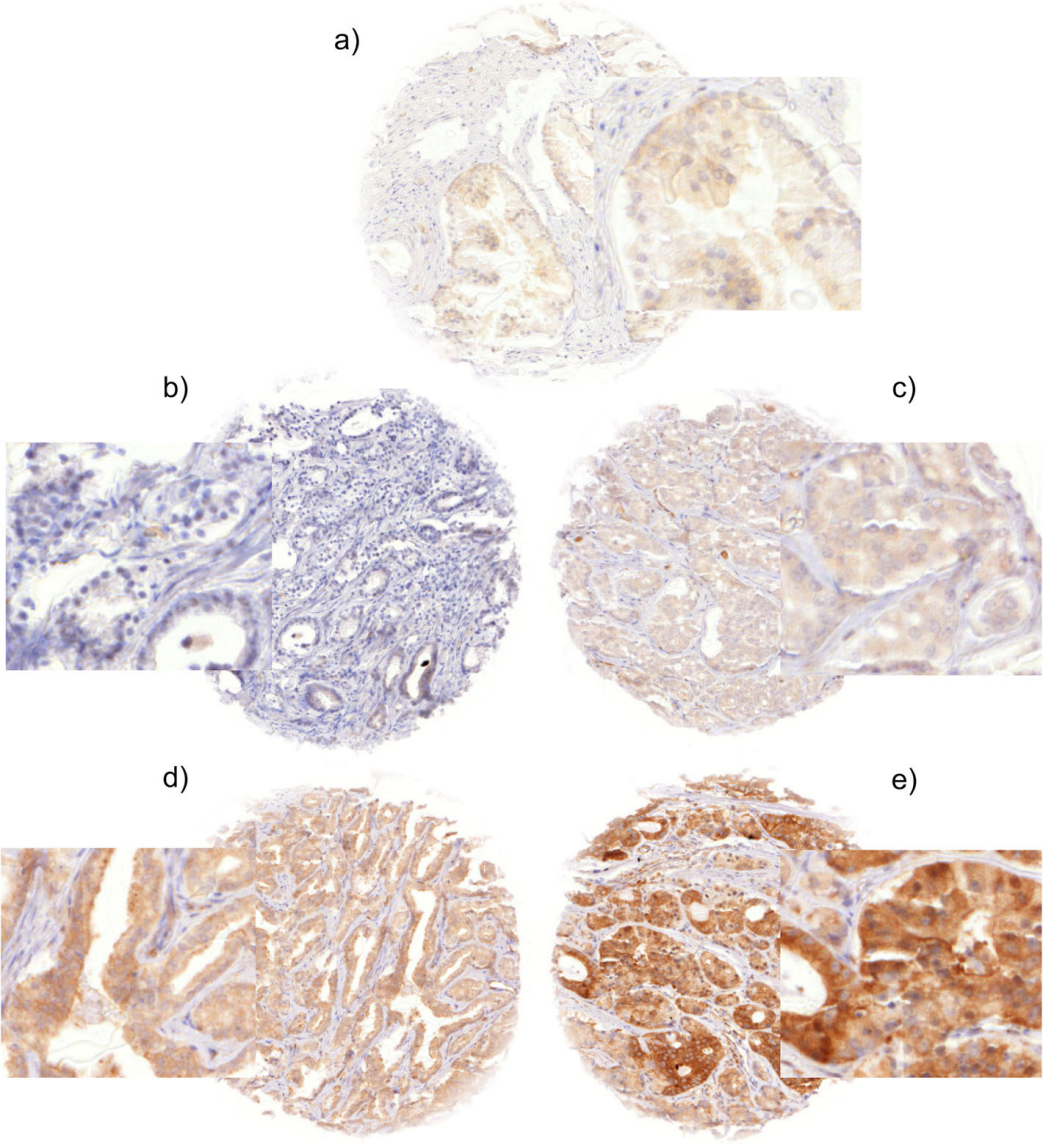
Representative images of PTPN12 staining in normal (**a**) and cancerous glands (**b-e**) with negative (**b**), weak (**c**), moderate (**d**) and strong (**e**) staining. Spot size is 600 μm at 100 / 400x magnification

Tissue microarray sections were stained in a single experiment. Slides were dewaxed and heated for 5 min at 121 °C in pH 9.0 antigen retrieval buffer. Primary antibody HPA007097 specific for PTPN12 (rabbit polyclonal antibody, dilution 1:450; Sigma-Aldrich, St. Louis, Missouri, USA) was applied at 37 °C for 60 min. This antibody was comprehensively validated externally (https://www.proteinatlas.org/ENSG00000127947-PTPN12/antibody#ICC) [38, 39]. Bound antibody was visualized with the EnVision Kit (Dako, Glostrup, Denmark). PTPN12 typically shows cytoplasmic staining of all tumor cells (100%) of a positive tissue spot with equal staining intensity. Thus, only staining intensity was recorded in a semi quantitative 4-step scale. ‘Negative’ was assigned if no detectable staining was present. ‘Strong’ was assigned to all tumors showing intense, dark brown staining. ‘Weak’ or ‘moderate’ was assigned to cancer showing staining intensities in between; e.g. as shown in Fig. 1. To rule out interobserver variability scoring was based on a single observer.

### Statistics

Contingency tables and the chi^2^-test were utilized to examine associations between molecular and histopathological tumor parameters. Kaplan-Meier curves were compared by the log-rank test to detect significant differences between groups. Cox proportional hazards regression analysis was performed to test for statistical independence between pathological, molecular and clinical variables. All calculations were performed with JMP 12 (SAS Institute Inc., NC, USA).

## Results

### Technical aspects

A total of 10,317 (76%) of the 13,660 arrayed tumor samples displayed interpretable PTPN12 staining. Non-informative cases (24%) were caused by lack of tissue at certain TMA spots or absence of unequivocal cancer cells.

### PTPN12 protein expression in normal and cancerous prostate tissues

In normal prostate epithelial cells, PTPN12 was negative or displayed a weak cytoplasmic immunostaining while basal cells frequently had a moderate positivity (Fig. 1). PTPN12 immunostaining was often more intense in cancers. It was considered negative in 28.9%, weak in 26.5%, moderate in 39.9%, and strong in 4.7% of cancers (Table 2). High level PTPN12 staining was associated with advanced pT category, high conventional and quantitative Gleason grade, and positive surgical margin status and to a higher likelihood for PSA recurrence (*p* < 0.0001 each).

**Table 2:** PTPN12 staining results of the primary tumor and prostate cancer phenotype in all cancers

Parameter	N	PTPN12 (%)				P
		Negative	Weak	Moderate	Strong	
All cancers	10,317	28.9	26.5	39.9	4.7	
Tumor stage						< 0.0001
pT2	6438	32.8	26.9	36.7	3.6	
pT3a	2385	24.2	25.7	44.6	5.5	
pT3b-pT4	1448	19.5	26.0	47.0	7.6	
Gleason grade						< 0.0001
≤ 3 + 3	1999	39.6	29.1	26.5	4.8	
3 + 4	5526	29.2	26.9	40.3	3.6	
3 + 4 Tert.5	444	26.4	26.1	44.4	3.2	
4 + 3	1030	20.8	26.0	47.0	6.2	
3 + 4 Tert.5	711	18.1	20.1	53.9	7.9	
≥ 4 + 4	599	18.9	23.9	48.7	8.5	
Quantitative Gleason grade						< 0.0001
≤ 3 + 3	1971	39.7	29.1	26.3	4.8	
3 + 4 ≤ 5%	1305	33.4	27.2	36.2	3.2	
3 + 4 6–10%	1288	31.4	26.8	38.5	3.3	
3 + 4 11–20%	1059	28.0	25.1	44.2	2.6	
3 + 4 21–30%	600	25.0	26.7	42.7	5.7	
3 + 4 31–49%	483	26.5	25.5	43.9	4.1	
3 + 4 Tert.5	323	28.2	28.2	41.8	1.9	
4 + 3 50–60%	400	22.0	23.5	49.0	5.5	
4 + 3 61–80%	345	20.0	25.2	51.0	3.8	
4 + 3 > 80%	93	19.4	25.8	43.0	11.8	
4 + 3 Tert.5	518	20.5	21.6	53.3	4.6	
≥ 4 + 4	406	20.4	25.6	48.3	5.7	
Lymph node metastasis						< 0.0001
N0	6081	27.0	26.4	41.9	4.8	
N+	718	17.4	22.0	53.5	7.1	
Preoperative PSA level (ng/ml)						0.0158
< 4	1222	25.1	26.1	42.7	6.1	
4–10	6084	29.4	26.8	39.6	4.2	
10–20	2146	29.7	25.4	39.7	5.1	
> 20	752	27.9	28.1	39.5	4.5	
Surgical margin						< 0.0001
Negative	8120	30.0	26.5	39.3	4.2	
Positive	1982	24.3	27.0	42.2	6.4	

**Fig. 2:**
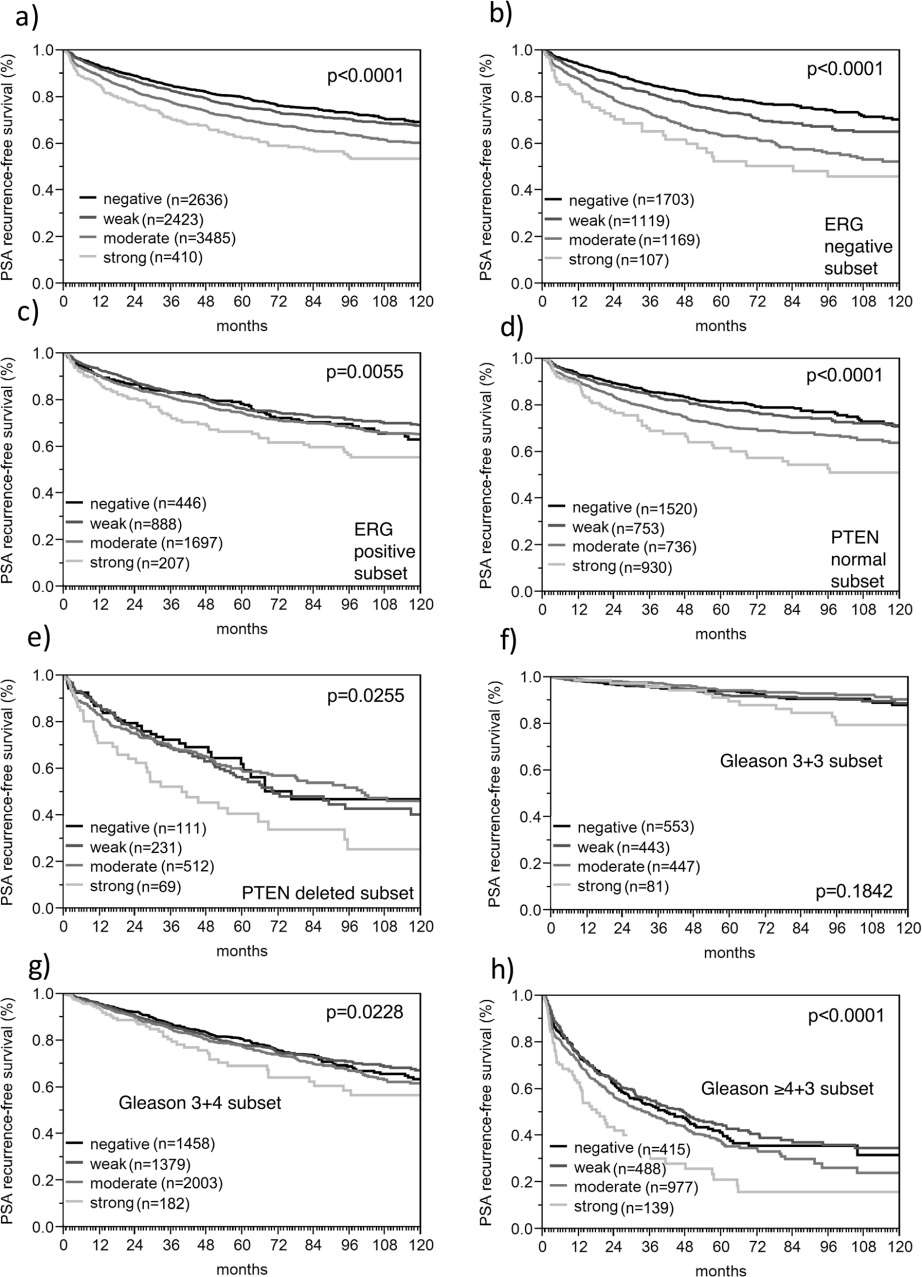
Association between PTPN12 expression and biochemical recurrence in (**a**) all cancers, (**b**) *ERG*-fusion negative cancers, (**c**) *ERG*-fusion positive cancers, (**d**) PTEN normal cancers, (**e**) PTEN deleted cancers, (**f**) Gleason grade 3 + 3, (**g**) Gleason grade 3 + 4 and (**h**) Gleason grade ≥ 4 + 3

It is of note that the prognostic impact of high PTPN12 staining (Fig. 2a) was also retained in PTEN deleted cancers (Fig. 2e) and in cancers with a Gleason 3 + 4 (Fig. 2g) or Gleason ≥4 + 3 (Fig. 2h). It disappeared in most of the quantitative Gleason categories (Additional file 1: Figure S1 b-g) and remained in the category with the highest percentage of Gleason 4 patterns (Additional file 1: Figure S1 h).

### PTPN12 and TMPRSS2:*ERG* fusion status

**Fig. 3:**
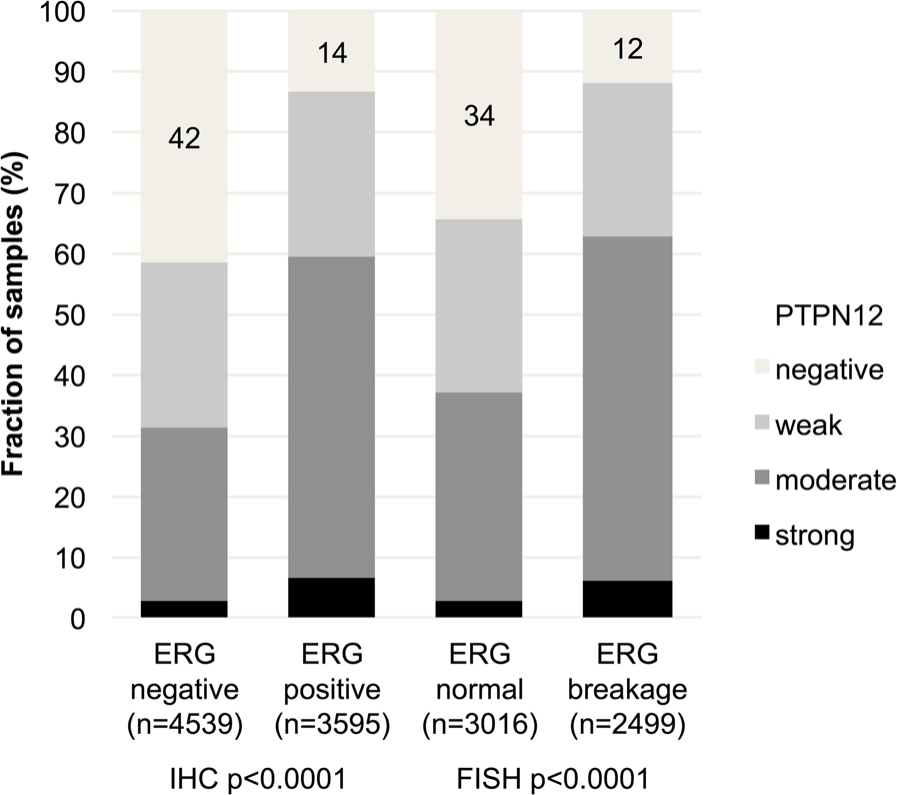
Association between PTPN12 staining and ERG-status in IHC and FISH analysis

ERG fusion status by FISH and by IHC was available from 5515 and 8134 tumors respectively (Fig. 3). Concordant results regarding the ERG status using IHC and FISH was obtained in 95.4% of cases. PTPN12 immunostaining was more prevalent in ERG fusion positive than in ERG wild type cancers. PTPN12 immunostaining was seen in 86.4% of ERG IHC positive and in only 58.4% of ERG IHC negative cancers (*p* < 0.0001). Because of these differences, all analyses comparing PTPN12 expression and tumor phenotype or prognosis were also performed in subgroups of ERG positive and negative cancers. This revealed a tighter relationship of high PTPN12 staining levels with unfavorable tumor features in ERG negative than in ERG positive cancers (Fig. 2b and c; Additional file 1: Tables S1 and S2). This was particularly evident for the relationship with PSA recurrence, which was striking in ERG negative (*p* < 0.0001, Fig. 2b) but much less strong in ERG positive cancers (*p* = 0.0055, Fig. 2c).

### PTPN12 and chromosomal deletions

**Fig. 4:**
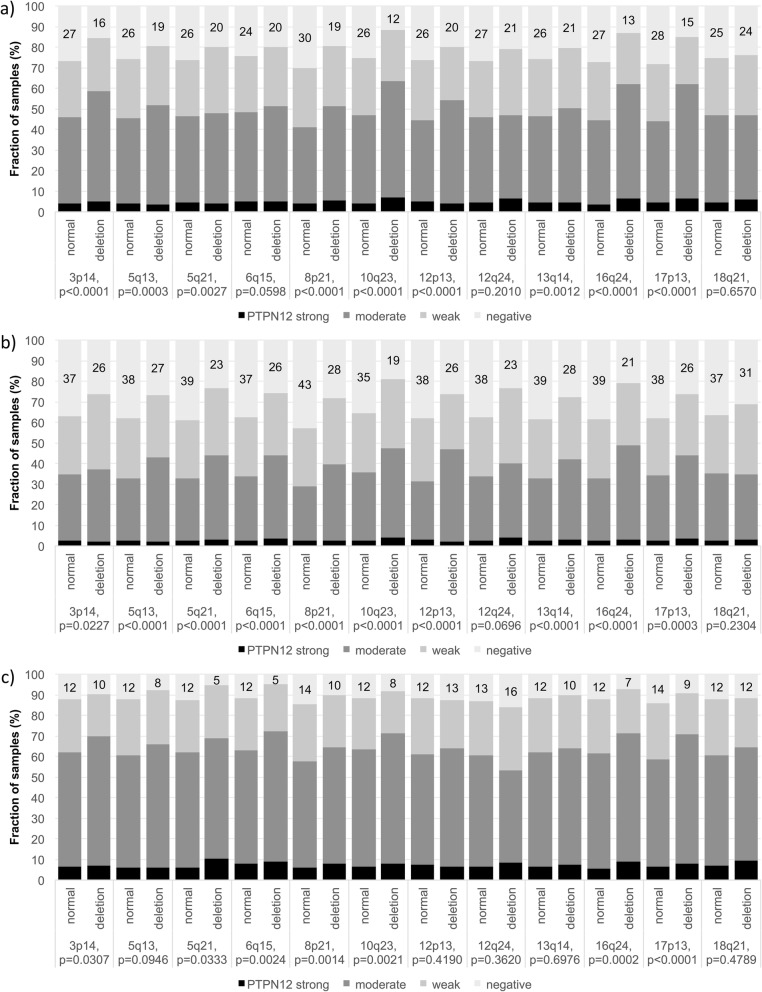
Association between PTPN12 staining and common chromosomal deletions in **a** all cancer, **b** in ERG negative cancers and **c** in ERG positive cancers

For all analyzed chromosomal regions, PTPN12 immunostaining was always stronger and more frequent in cases of deletion (Fig. 4a). This was particularly evident in the subgroup of ERG negative cancers where this difference was statistically significant for 9 of 12 deletions (*p* < 0.0005 each, Fig. 4b). In ERG positive cancers, a statistically significant difference was still seen for 7 of 12 analyzed deletions (*p* < 0.05 each, Fig. 4c).

### PTPN12, tumor cell proliferation and HER2 immunostaining

High levels of PTPN12 staining were linked to increased cell proliferation as determined by the Ki67-labeling index (Ki67LI). The average Ki67LI increased from 1.82 in PTPN12 negative cancers to 3.61 in cancers with strong PTPN12 staining (Table 3). This association was independent from Gleason score as it held true in all subgroups with high significance (*p* < 0.0001 each) except for Gleason score ≥ 4 + 3 (*p* < 0.0047).

**Table 3:** Association between PTPN12 expression and Ki67-labeling index

Gleason (*p*-value)	PTPN21	N	Ki67 LI (mean ± SEM)
All (*p* < 0.0001)	Negative	1673	1.82 ± 0.06
	Weak	1518	2.79 ± 0.07
	Moderate	2103	3.36 ± 0.06
	Strong	198	3.61 ± 0.18
≤3 + 3 (*p* < 0.0001)	Negative	492	1.50 ± 0.09
	Weak	362	1.98 ± 0.11
	Moderate	332	2.39 ± 0.11
	Strong	49	2.50 ± 0.29
3 + 4 *p* < 0.0001	Negative	926	1.59 ± 0.07
	Weak	863	2.58 ± 0.08
	Moderate	1301	3.10 ± 0.06
	Strong	96	2.67 ± 0.23
4 + 3 (*p* < 0.0001)	Negative	189	1.8676 ± 0.26
	Weak	223	2.9945 ± 0.24
	Moderate	350	3.7877 ± 0.19
	Strong	38	3.4073 ± 0.57
≥4 + 3 (*p* = 0.0047)	Negative	54	1.5949 ± 1.5949
	Weak	65	3.8142 ± 3.8142
	Moderate	107	4.1036 ± 4.1036
	Strong	14	4.3912 ± 4.3912

**Fig. 5:**
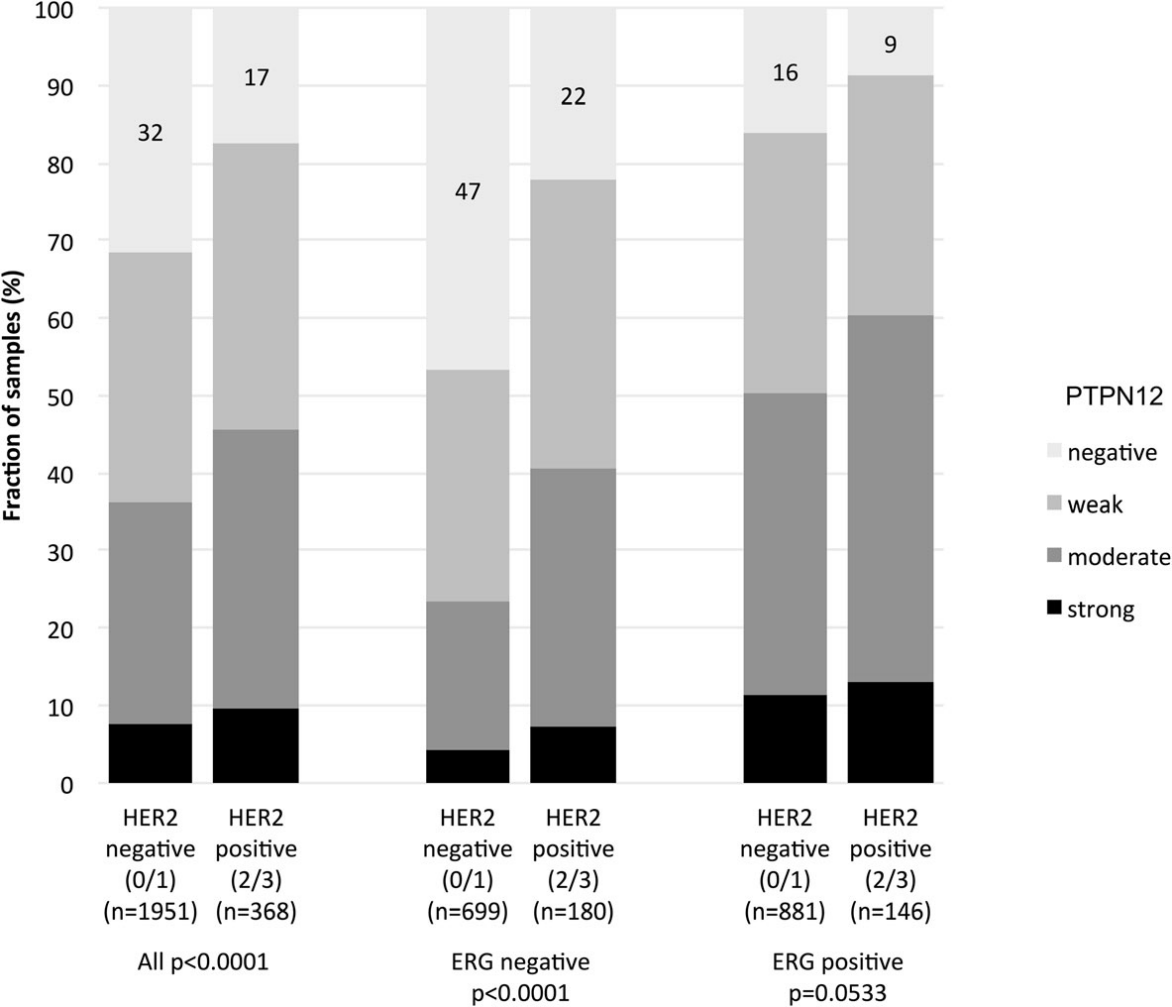
PTPN12 staining and HER2 expression in all cancers, the ERG negative, and the ERG positive subset

PTPN12 staining was significantly associated with the expression of HER2 protein (Fig. 5). Negative PTPN12 staining was seen in 32% of HER2 negative cancers and in 17% of HER2 positive cancers. The same effect was seen in both ERG subsets.

### Multivariate analysis

Four different models were analyzed (Additional file 1: Table S3): Scenario 1 included the postoperatively available parameters pT, pN, surgical margin status, preoperative PSA value and prostatectomy Gleason grade. Scenario 2 excluded pN, because the lymph node dissection is not standardized and may introduce a bias towards high-grade cancers. Scenario 3 was a mix of pre- and postoperative parameters (PTPN12 staining, preoperative serum PSA, clinical tumor stage (cT) and the prostatectomy Gleason grade). Since it is well documented that sampling differences lead to up-grading of the postoperative Gleason grades in 36% of cases [40], this parameter was replaced by the original preoperative biopsy Gleason grade in Scenario 4. These analyses identified PTPN12 as an independent prognostic feature in all 4 scenarios, if the entire cohort or the subgroup of ERG negative cancers was considered (*p* < 0.0005 each). Independent prognostic impact, although weaker, was also seen in the ERG positive cancer subset (*p* < 0.005 each). The hazard ratio for PSA recurrence after radical prostatectomy for strong versus negative PTPN12 expression was in the univariate model a weak 1.85 for all cancers and a moderate 2.50 in the ERG negative subset as compared with 6.01 for the Gleason grade at biopsy (Table 4).

**Table 4:** Cox proportional hazards for PSA recurrence-free survival after prostatectomy of established preoperative prognostic parameter and PTPN12 expression

Variable	Univariable analysis	Multivariable analysis
Gleason grade biopsy		
≥ 4 + 4 vs. ≤3 + 3	6.01 (5.41–6.66) ***	4.21 (3.71–4.79) ***
Preoperative PSA-level (ng/μl)		
> 20 vs. < 4	5.12 (4.46–5.89) ***	3.14 (2.61–3.80) ***
cT-stage		
T2c vs. T1c	3.95 (3.24–4.76) ***	2.08 (1.66–2.58) ***
PTPN12 expression		
Strong vs. negative	1.85 (1.53–2.23) ***	1.71 (1.40–2.07) ***
ERG negative subset	2.50 (1.82–3.35) ***	2.28 (1.65–3.09) ***
ERG positive subset	1.51 (1.23–2.02) *	1.37 (1.01–1.85) *

Confidence interval (95%) in brackets; asterisk indicate significance level: * *p* ≤ 0.05, ** *p* ≤ 0.001, *** *p* ≤ 0.0001; ERG ETS-related gene

## Discussion

These data identify high PTPN12 expression as an independent predictor of poor prognosis in prostate cancer.

That PTPN12 immunostaining increased from normal to cancerous epithelial cells in combination with the marked further increase of PTPN12 expression with advanced tumor stage and high Gleason grade, demonstrates that elevated PTPN12 expression parallels tumor development and progression in a fraction of prostate cancers. The striking prognostic role of high PTPN12 expression being independent of all established prognostic features available before and after prostatectomy in our study on 13,660 cancers was not expected. Both functional data from prostate cancer cell lines [20] and earlier reports on PTPN12 down regulation in other cancer types [10–19] suggest a tumor suppressor function of PTPN12. However, that tumor suppressor genes are overexpressed in cancer cells is not uncommon. For example, the tumor suppressor p16 is markedly up regulated in cells infected by human papilloma virus in an attempt to compensate for disrupted p53 and rb pathways [41, 42]. P16 expression is so massive in affected cells, that p16 expression analysis can be used in HPV associated neoplasia in routine diagnostic [43, 44]. Moreover, it is well possible that the causes and consequences of PTPN12 overexpression differ between different cancer types. Some studies analyzing the prognostic value of PTPN12 in small cohorts of up to 250 patients report a positive correlation of increased PTPN12 expression and outcome in non small cell lung cancer [18], breast cancer [45] and squamous cell carcinoma [14], whereas Zhangyuan et al. found a contrary result in their study in at least one subgroup of non-hepatitis B-positive patients with hepatocellular carcinoma [11]. At present, there is no mechanistic explanation for these findings. However, similar observations have been reported from the tumor suppressor checkpoint kinase 2 (CHK2), a protein interacting with p53 and BRCA1. Both reduced and increased CHK2 expression has been described in different tumor types to be associated with poor patient prognosis [46–48]. The largest study investigating the prognostic role of CHK2 expression on more than 1000 well characterized breast cancers failed to show a prognostic impact of CHK2 expression in all cancers but revealed associations of high CHK2 expression with poor patient outcome in p53 positive and ER negative cancers while low CHK2 expression was linked to poor prognosis in ER positive cancers [49].

The TMA used in this study had earlier been utilized for dozens of studies evaluating the clinical relevance of molecular features in prostate cancer [50]. This led to an accumulation of relevant molecular information for our patient cohort that can potentially be utilized to hypothesize on the possible functional role of new genes of interest. For the purpose of this study, we compared PTPN12 expression with TMPRSS2:ERG fusion because this is the most common molecular alteration in prostate cancer [51], 12 different chromosomal deletions representing the next most common genomic alterations in prostate cancer [52], the Ki67 labeling index because of its pivotal role in cancer aggressiveness [53], and immunohistochemical HER2 expression because of the earlier well described interaction with PTPN12 [3, 54]. The significant association of PTPN12 and HER2 expression seen in our patients therefore fits well. *TMPRSS2:ERG* fusions occur in about 50% of prostate cancers and result in a permanent expression of the transcription factor ERG. ERG activation by itself lacks prognostic relevance [25] but modulates the expression of more than 1600 genes in affected cells [55]. Our data identify PTPN12 protein as another protein whose expression was increased in ERG positive compared to ERG negative cancers.

That the prognostic role of PTPN12 was more striking in ERG negative and somewhat less prominent in ERG positive cancers fits with the observation, that many molecular features that show different prevalence in ERG positive and ERG negative cancers have a different impact on patient prognosis in these subgroups. For example, the prognostic impact of SOX9 [56], SENP1 [57] and mTOR [58] was limited to ERG positive cancers while FOXA1 [59], MTCO2 [60] and FOXP2 [61] were only prognostic in ERG negative cancers. It is well conceivable that differences in the cellular microenvironment with more than 1600 dysregulated genes in ERG activated cancers impact the biological effect of molecular features such as PTPN12. Dependency of the prognostic impact of biomarkers on other specific molecular tumor features is likely to constitute a significant challenge for the development of prognostic prostate cancer tests.

Most chromosomal deletions are linked to either positive or negative ERG status [28–30, 62]. Molecular features that are also linked to the ERG status, such as PTPN12, are thus expected to show statistically significant associations with ERG dependent deletions. That a separate analysis of subgroups identified significant relationship between high PTPN12 expression and 10 of 12 deletions in ERG negative and of 7 of 12 deletions in ERG positive cancers shows, however, that elevated PTPN12 levels preferentially occur under conditions linked to genomic instability in prostate cancers. That none of the deletions examined in this study was more prominently linked to PTPN12 expression argues against a relevant functional relationship of PTPN12 with genes impacted by these deletions. It seems more likely that the PTPN12 up regulation results from a general response to genetic instability. One of PTPN12s substrates, WASP [63], mediates homology-direct repair together with Arp2/3 in DNA double-strand breaks [64] and could therefore be a conceivable link to PTPN12 overexpression. Also Tang et al. were able to demonstrate that suppression of FAK1, also a target of PTPN12-dephosphorylation [65], leads to activation of DNA repair in lung cancer [66].

Besides the two mentioned, 16 more substrates of PTPN12 are currently known including HER2, PYK2, PSTPIP, p130CAS/BCAR1, paxillin, Shc, catenin, c-Abl, ArgBP2, CAKß and members of the Rho proteins [3, 9, 63, 65, 67–74]. Several of these genes play a particular role in the growth controlling EGFR-pathway, which fits well to the markedly elevated Ki67 LI in cancers with high PTPN12 expression. Especially FAK1 is of particular interest in this context. For example, in colonic carcinoma, Fonar and Frank were able to show that FAK is in connection with the Wnt signaling pathway at several sites [75]. In particular, cell cycle control is regulated by transcriptional control of cyclin D1 via FAK. In turn, the Wnt signaling pathway is known to be massively up regulated in ERG translocated prostate carcinomas [76]. This fits with our observations suggesting that this pathway is strongly driven in ERG positive tumors.

This study suggests that PTPN12 expression may represent a useful prognostic biomarker in prostate cancer. This is not only illustrated by the statistical independence of all established prognostic parameters, even if parameters are included that are – such as pT and pN – unavailable at the time, when therapeutic decisions are taken. Moreover, PTPN12 retained prognostic impact in molecularly defined high risk groups such as in PTEN deleted cancers and in some morphologically defined high-risk groups such as in Gleason 3 + 4 cancers. That PTPN12 expression analysis was not better than Gleason grading does not compromise the potential for PTPN12 expression analysis, however. Although Gleason grading is a very powerful statistical parameter, it suffers from notorious interobserver heterogeneity, which is in the range of 40% [77, 78]. Accordingly, there is not only a need for better predictors of PCA aggressiveness than the established ones but also for more reproducible ones. Molecular analysis may, thus, help to improve standardization of prognosis assessment in the future.

## Conclusions

This study identifies PTPN12 expression measurement as a valuable prognostic marker in prostate cancer. PTPN12 analysis, either alone or in combination might be of clinical utility in the prognostic assessment of prostate cancers.

## Supplementary information

**Additional file 1: Table S1.** Association between protein tyrosine phosphatase non-receptor 12 (PTPN12) staining results and prostate cancer phenotype in *ERG* fusion *negative* tumors. **Table S2.** Association between protein tyrosine phosphatase non-receptor 12 (PTPN12) staining results and prostate cancer phenotype in *ERG* fusion *positive* tumors. **Table S3.** Multivariate analysis including PTPN12 expression in all cancers, ERG negative and ERG positive cancers. **Figure S1.** PTPN12 expression (negative vs. strong) and biochemical recurrence in (a) classic Gleason grade (b) < 5% Gleason 4, (c) 6–10% Gleason 4, (d) 11–20% Gleason 4, (e) 21–30% Gleason 4, (f) 31–49% Gleason 4, (g) 50–60% Gleason 4, (h) 61–100% Gleason 4.

## Data Availability

The data supporting the findings of this study are available from the corresponding author upon reasonable request.

## Abbreviations

CHD1: Chromodomain-Helicase-DNA-Binding Protein 1
FISH: Fluorescence in-situ hybridization
FOXP1: Forkhead box protein P1
IHC: Immunohistochemistry
MAP3K7: Mitogen-Activated Protein Kinase Kinase Kinase 7
PSA: Prostate specific antigen
PTEN: Phosphatase and tensin homolog
PTPN12: Protein phosphatase non-receptor 12
TMA: Tissue microarray
TMPRSS2:ERG: Transmembrane protease, serine 2: ETS-related gene fusion
