# Supplementary material for: High-level expression of protein tyrosine phosphatase non-receptor 12 is a strong and independent predictor of poor prognosis in prostate cancer
Source: BMC Cancer. 2019 Oct 12;19:944. doi: 10.1186/s12885-019-6182-3 (PMC6790047; doi:10.1186/s12885-019-6182-3)
Supplement: Supplementary file 1 — Additional file 1: Table S1. Association between protein tyrosine phosphatase non-receptor 12 (PTPN12) staining results and prostate cancer phenotype in ERG fusion negative tumors. Table S2. Association between protein tyrosine phosphatase non-receptor 12 (PTPN12) staining results and prostate cancer phenotype in ERG fusion positive tumors. Table S3. Multivariate analysis including PTPN12 expression in all cancers, ERG negative and ERG positive cancers. Figure S1. PTPN12 expression (negative vs. strong) and biochemical recurrence in (a) classic Gleason grade (b) < 5% Gleason 4, (c) 6–10% Gleason 4, (d) 11–20% Gleason 4, (e) 21–30% Gleason 4, (f) 31–49% Gleason 4, (g) 50–60% Gleason 4, (h) 61–100% Gleason 4. [file 12885_2019_6182_MOESM1_ESM.docx]

**Table S1**: Association between protein tyrosine phosphatase non-receptor 12 (PTPN12) staining results and prostate cancer phenotype in *ERG* fusion *negative* tumors

| **Parameter** |  | **PTPN12 (%)** | | | |  |
| --- | --- | --- | --- | --- | --- | --- |
|  | **N** | **Negative** | **Weak** | **Moderate** | **Strong** | **P** |
| **All cancers** | 4,539 | 41.6 | 27.3 | 28.6 | 2.5 |  |
|  |  |  |  |  |  |  |
| **Tumor stage** |  |  |  |  |  | <0.0001 |
| pT2 | 3,005 | 45.6 | 26.7 | 25.7 | 2.1 |  |
| pT3a | 923 | 38.5 | 27.6 | 31.4 | 2.5 |  |
| pT3b-pT4 | 596 | 26.5 | 30.0 | 38.8 | 4.7 |  |
|  |  |  |  |  |  |  |
| **Gleason grade** |  |  |  |  |  | <0.0001 |
| ≤3+3 | 910 | 53.8 | 25.5 | 18.4 | 2.3 |  |
| 3+4 | 2,406 | 43.1 | 27.9 | 27.0 | 2.0 |  |
| 3+4 Tert.5 |  | 40.1 | 28.3 | 31.0 | 0.5 |  |
| 4+3 | 486 | 29.2 | 27.8 | 39.1 | 3.9 |  |
| 3+4 Tert.5 |  | 28.4 | 25.5 | 41.7 | 4.4 |  |
| ≥4+4 | 276 | 24.6 | 28.6 | 42.0 | 4.7 |  |
|  |  |  |  |  |  |  |
| **quant Gleason grade** |  |  |  |  |  | <0.0001 |
| ≤3+3 | 910 | 53.8 | 25.5 | 18.4 | 2.3 |  |
| 3+4 ≤5% | 649 | 46.7 | 27.7 | 23.9 | 1.7 |  |
| 3+4 6-10% | 621 | 46.4 | 27.2 | 25.0 | 1.4 |  |
| 3+4 11-20% | 523 | 40.2 | 28.1 | 30.4 | 1.3 |  |
| 3+4 21-30% | 283 | 38.2 | 27.2 | 30.0 | 4.6 |  |
| 3+4 31-49% | 241 | 39.8 | 27.0 | 31.1 | 2.1 |  |
| 3+4 Tert.5 | 187 | 40.1 | 28.3 | 31.0 | 0.5 |  |
| 4+3 50-60% | 207 | 30.9 | 26.6 | 39.6 | 2.9 |  |
| 4+3 61-80% | 180 | 26.1 | 28.3 | 41.7 | 3.9 |  |
| 4+3 >80% | 49 | 24.5 | 22.4 | 40.8 | 12.2 |  |
| 4+3 Tert.5 | 271 | 28.4 | 25.5 | 41.7 | 4.4 |  |
| ≥4+4 | 243 | 24.3 | 26.3 | 44.9 | 4.5 |  |
|  |  |  |  |  |  |  |
| **Lymph node metastasis** |  |  |  |  |  | <0.0001 |
| N0 | 2,640 | 38.9 | 28.0 | 30.3 | 2.8 |  |
| N+ | 270 | 25.2 | 26.7 | 44.4 | 3.7 |  |
|  |  |  |  |  |  |  |
| **Preop. PSA level (ng/ml)** |  |  |  |  |  | 0.0084 |
| <4 | 479 | 34.9 | 26.7 | 34.2 | 4.2 |  |
| 4-10 | 2,656 | 42.6 | 27.7 | 27.6 | 2.1 |  |
| 10-20 | 990 | 43.3 | 26.2 | 27.8 | 2.7 |  |
| >20 | 371 | 38.3 | 28.0 | 30.7 | 3.0 |  |
|  |  |  |  |  |  |  |
| **Surgical margin** |  |  |  |  |  | 0.0014 |
| negative | 3,590 | 42.9 | 26.7 | 28.1 | 2.3 |  |
| positive | 866 | 35.8 | 30.0 | 30.9 | 3.2 |  |

**Table S2**: Association between protein tyrosine phosphatase non-receptor 12 (PTPN12) staining results and prostate cancer phenotype in *ERG* fusion *positive* tumors

| **Parameter** |  | **PTPN12 (%)** | | | |  |
| --- | --- | --- | --- | --- | --- | --- |
|  | **N** | **Negative** | **Weak** | **Moderate** | **Strong** | **P** |
| **All cancers** | 3,595 | 13.6 | 26.8 | 53.2 | 6.3 |  |
|  |  |  |  |  |  |  |
| **Tumor stage** |  |  |  |  |  | 0.0033 |
| pT2 | 2,090 | 14.9 | 27.8 | 51.7 | 5.6 |  |
| pT3a | 978 | 10.9 | 25.9 | 56.6 | 6.5 |  |
| pT3b-pT4 | 510 | 13.1 | 24.7 | 53.3 | 8.8 |  |
|  |  |  |  |  |  |  |
| **Gleason grade** |  |  |  |  |  | <0.0001 |
| ≤3+3 | 710 | 19.0 | 33.8 | 39.0 | 8.2 |  |
| 3+4 | 2,062 | 13.0 | 26.2 | 55.3 | 5.4 |  |
| 3+4 Tert.5 | 111 | 9.0 | 26.1 | 60.4 | 4.5 |  |
| 4+3 | 362 | 9.9 | 24.6 | 56.9 | 8.6 |  |
| 3+4 Tert.5 | 204 | 9.3 | 16.2 | 70.6 | 3.9 |  |
| ≥4+4 | 144 | 13.9 | 22.9 | 52.8 | 10.4 |  |
|  |  |  |  |  |  |  |
| **quant Gleason grade** |  |  |  |  |  | <0.0001 |
| ≤3+3 | 710 | 19.0 | 33.8 | 39.0 | 8.2 |  |
| 3+4 ≤5% | 502 | 14.9 | 26.5 | 53.2 | 5.4 |  |
| 3+4 6-10% | 538 | 11.5 | 25.7 | 57.8 | 5.0 |  |
| 3+4 11-20% | 436 | 13.3 | 20.0 | 62.6 | 4.1 |  |
| 3+4 21-30% | 270 | 10.7 | 24.4 | 58.1 | 6.7 |  |
| 3+4 31-49% | 199 | 11.1 | 24.6 | 56.8 | 7.5 |  |
| 3+4 Tert.5 | 111 | 9.0 | 26.1 | 60.4 | 4.5 |  |
| 4+3 50-60% | 155 | 9.7 | 18.7 | 61.9 | 9.7 |  |
| 4+3 61-80% | 135 | 8.1 | 21.5 | 65.9 | 4.4 |  |
| 4+3 >80% | 31 | 16.1 | 29.0 | 45.2 | 9.7 |  |
| 4+3 Tert.5 | 204 | 9.3 | 16.2 | 70.6 | 3.9 |  |
| ≥4+4 | 120 | 11.7 | 24.2 | 55.8 | 8.3 |  |
|  |  |  |  |  |  |  |
| **Lymph node metastasis** |  |  |  |  |  | 0.3295 |
| N0 | 2,082 | 12.2 | 26.5 | 55.1 | 6.2 |  |
| N+ | 244 | 12.3 | 21.3 | 60.2 | 6.1 |  |
|  |  |  |  |  |  |  |
| **Preop. PSA level (ng/ml)** |  |  |  |  |  | 0.5617 |
| <4 | 498 | 13.3 | 26.5 | 53.4 | 6.8 |  |
| 4-10 | 2,176 | 14.3 | 26.5 | 53.2 | 6.0 |  |
| 10-20 | 646 | 11.0 | 26.2 | 55.9 | 7.0 |  |
| >20 | 231 | 13.9 | 30.3 | 50.2 | 5.6 |  |
|  |  |  |  |  |  |  |
| **Surgical margin** |  |  |  |  |  | 0.0272 |
| negative | 2,805 | 14.1 | 27.1 | 53.1 | 5.8 |  |
| positive | 719 | 11.3 | 26.7 | 53.7 | 8.3 |  |

**Table S3:** Multivariate analysis including PTPN12 expression in all cancers, ERG negative and ERG positive cancers

| **Subset** | **Scen-ario** | **N Gleason / N quantitative Gleason** | **p –value Gleason (p-value quantitative Gleason)** | | | | | | | |
| --- | --- | --- | --- | --- | --- | --- | --- | --- | --- | --- |
|  |  |  | **Preoperative PSA-level** | **pT-stage** | **cT-stage** | **Gleason grade prostatectomy** | **Gleason grade biopsy** | **pN-stage** | **R-stage** | **PTPN12-expression** |
| **All** | 1 | 5,667 / 4,750 | <0.0001 (<0.0001) | <0.0001 (<0.0001) | - | <0.0001 (<0.0001) | - | <0.0001 (0.0002) | 0.0021 (0.006) | <0.0001 (<0.0001) |
|  | 2 | 8,733 / 7,712 | <0.0001 (<0.0001) | <0.0001 (<0.0001) | - | <0.0001 (<0.0001) | - | - | <0.0001 (<0.0001) | <0.0001 (<0.0001) |
|  | 3 | 8,637 / 7,641 | <0.0001 (<0.0001) | - | <0.0001 (<0.0001) | <0.0001 (<0.0001) | - | - | - | <0.0001 (<0.0001) |
|  | 4 | 8,536 | <0.0001 | - | <0.0001 | - | <0.0001 | - | - | <0.0001 |
| **ERG-negative** | 1 | 2,552 | <0.0001 | <0.0001 | - | <0.0001 | - | 0.0006 | 0.3486 | <0.0001 |
|  | 2 | 3,989 | <0.0001 | <0.0001 | - | <0.0001 | - | - | 0.0222 | <0.0001 |
|  | 3 | 3,952 | <0.0001 | - | <0.0001 | <0.0001 | - | - | - | <0.0001 |
|  | 4 | 3,908 | <0.0001 | - | <0.0001 | - | <0.0001 | - | - | <0.0001 |
| **ERG-positive** | 1 | 2,039 | 0.0026 | <0.0001 | - | <0.0001 | - | 0.0566 | 0.0049 | 0.0102 |
|  | 2 | 3,161 | 0.0002 | <0.0001 | - | <0.0001 | - | - | <0.0001 | 0.0134 |
|  | 3 | 3,099 | <0.0001 | - | <0.0001 | <0.0001 | - | - | - | 0.0195 |
|  | 4 | 3,054 | <0.0001 | - | <0.0001 | - | <0.0001 | - | - | 0.0295 |

Scenario 1 combines postoperatively available parameter. Scenario 2 excludes the lymph node (pN) stage. Scenario 3 is a mix of post- and preoperative parameters. Scenario 4 includes the preoperative parameters (preoperative Gleason grade obtained on the original biopsy, clinical tumor (cT) stage, and preoperative PSA). Overall p-values are uncorrected for multiple comparisons.


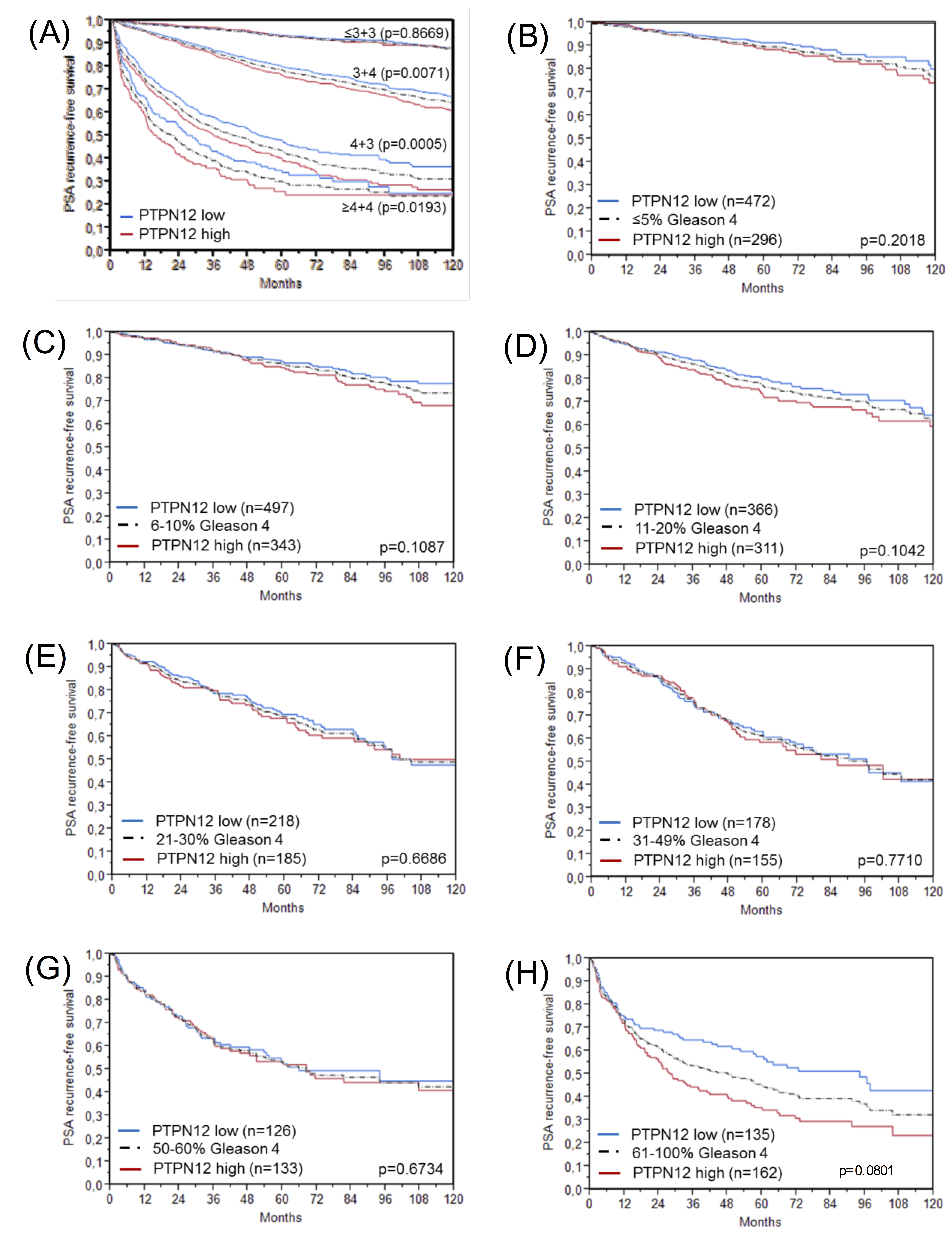


**Fig. S1.** PTPN12 expression (negative versus strong) and biochemical recurrence in (**a**) classic Gleason grade (**b**) <5% Gleason 4, (**c**) 6-10% Gleason 4, (**d**) 11-20% Gleason 4, (**e**) 21-30% Gleason 4, (**f**) 31-49% Gleason 4, (**g**) 50-60% Gleason 4, (**h**) 61-100% Gleason 4
